# A novel approach for predicting microbe-disease associations by bi-random walk on the heterogeneous network

**DOI:** 10.1371/journal.pone.0184394

**Published:** 2017-09-07

**Authors:** Shuai Zou, Jingpu Zhang, Zuping Zhang

**Affiliations:** School of Information Science and Engineering, Central South University, Changsha, Hunan, China; Texas A&M University College Station, UNITED STATES

## Abstract

Since the microbiome has a significant impact on human health and disease, microbe-disease associations can be utilized as a valuable resource for understanding disease pathogenesis and promoting disease diagnosis and prognosis. Accordingly, it is necessary for researchers to achieve a comprehensive and deep understanding of the associations between microbes and diseases. Nevertheless, to date, little work has been achieved in implementing novel human microbe-disease association prediction models. In this paper, we develop a novel computational model to predict potential microbe-disease associations by bi-random walk on the heterogeneous network (BiRWHMDA). The heterogeneous network was constructed by connecting the microbe similarity network and the disease similarity network via known microbe-disease associations. Microbe similarity and disease similarity were calculated by the Gaussian interaction profile kernel similarity measure; moreover, a logistic function was applied to regulate disease similarity. Additionally, leave-one-out cross validation and 5-fold cross validation were implemented to evaluate the predictive performance of our method; both cross validation methods performed well. The leave-one-out cross validation experiment results illustrate that our method outperforms other previously proposed methods. Furthermore, case studies on asthma and inflammatory bowel disease prove the favorable performance of our method. In conclusion, our method can be considered as an effective computational model for predicting novel microbe-disease associations.

## Introduction

There are a large number of microbes in the human body. Research indicates that approximately 90% of the cells in and on the human body are microbial cells [1]. These microbes, including bacteria, eukaryotes, archaea and viruses, reside in and on different body surfaces such as the mouth, skin, vagina and gut, with the vast majority residing in the gastrointestinal tract [2]. These microbes make up an important part of the human body. Recently, due to the impressive advances in metagenomics and metatranscriptomics tools, scientists have begun earnestly investigating the human microbiome. For example, the Human Microbiome Project (HMP) was recently launched to explore microbial communities and their relationships with human hosts [1]. The study found that the interaction between human microbiome and cells would affect human health and contribute to the pathogenesis of various diseases [3]. On the one hand, the relationship between humans and the microbiome is symbiotic and mutualistic. For instance, the gut microbiome advances nutrition and energy harvest by fermenting food components that cannot be digested by the host [4]. In addition, the microbiome can help develop the immune system [5, 6], maintain homeostasis [7], and protect against pathogens [8]. On the other hand, there is strong evidence that some microbiomes may lead to various diseases. Recent studies have discovered the associations between body microbiomes and ailments such as cancer [9], diabetes [10, 11], obesity [12–14] and kidney stones [15]. Thus, it is imperative for researchers to achieve a comprehensive understanding of the associations between microbes and diseases, which would not only help determine disease pathogenesis, but also boost disease diagnosis and therapy.

Though some computational methods have recently been proposed to study microorganisms and human diseases [16–18], little work has been undertaken to advance human microbe-disease association prediction models. Until 2016, Ma et al. built the Human Microbe-Disease Association Database (HMDAD) by collecting microbe-disease association data from 61 previous published studies, providing a valuable informational resource for investigating microbe-disease associations. Based on the freely available data, several network based prediction methods have been proposed to achieve microbe-disease association inference. Shen et al. developed RWRHMDA, which applies a random walk with restart algorithm on the heterogeneous network to rank candidate microbes for a specific disease [19]. Chen et al. proposed KATZHMDA to infer potential disease-related microbes by integrating walks of different lengths in the heterogeneous network [20]. Huang et al. introduced PBHMDA to obtain the prediction scores of each candidate microbe-disease pair by evaluating all paths between a microbe and a disease [21]. Meanwhile, during the last few years, the bi-random walk algorithm has been widely used in the field of bioinformatics to address biomedical problems [22–26]. Inspired by its superior performance, we apply bi-random walk algorithm to the study of human microbe-disease associations in the present study. It is a global strategy that explores the missing microbe-disease associations simultaneously, and can predict novel related microbes for diseases without any known associated microbe information.

More specifically, we present a novel computational approach that executes a bi-random walk algorithm on the heterogeneous network to predict potential microbe-disease associations (BiRWHMDA). Based on Gaussian interaction profile kernel similarity and logistic function transformation, we constructed the microbe similarity network and the disease similarity network. Subsequently, the heterogeneous network was constructed by connecting the microbe similarity network and the disease similarity network using the known microbe-disease associations. Then, the bi-random walk algorithm was executed on the heterogeneous network to predict potential microbe-disease associations. Cross validation frameworks are implemented to evaluate the performance of BiRWHMDA. The AUC (the area under of ROC curve) values were 0.8964 and 0.8808 in leave-one-out cross validation (LOOCV) and 5-fold cross validation, respectively. Experiment results of LOOCV demonstrate that our method outperforms other previously proposed methods. Furthermore, case studies of asthma and inflammatory bowel disease (IBD) also demonstrate the favorable performance of our method in predicting novel microbe-disease associations. In summary, BiRWHMDA can be considered as an effective predictive tool for potential microbe-disease associations.

## Materials and methods

### Dataset

The dataset used in this study (S1 File) was downloaded from the newly built Human Microbe-Disease Association Database (HMDAD, http://www.cuilab.cn/hmdad), which collects human microbe-disease association data from 61 previously published studies. Presently, HMDAD possesses 483 verified microbe-disease association records between 292 microbes and 39 diseases. Here, the microbes are curated at the genus level [27]. However, the set had several duplicate associations; after removing the duplications, we acquired 450 distinct associations and then constructed an adjacency matrix *A* of the microbe-disease association network. *A*(*i*,*j)* is equal to 1 if there is a known association between disease *d(i)* and microbe *m(j)*; otherwise, the appropriate coding is 0 [20].

### Microbe similarity

To construct the heterogeneous network, the microbe similarity network and the disease similarity network should be separately constructed. Further, we needed to ascertain the similarity between each microbe-microbe pair and each disease-disease pair. In this work, we apply the Gaussian interaction profile kernel similarity measure to determine microbe similarity and disease similarity [28–36].

Based on the assumption that similar microbes are more likely to show a similar interaction and non-interaction pattern with diseases, Gaussian interaction profile kernel similarity for microbes can be calculated from the known microbe-disease association network [33]. The microbe interaction profile *m(i)* is a binary vector encoding the presence or absence of the associations with each disease in the known microbe-disease association network, defined as the *ith* column of the adjacency matrix *A* of the microbe-disease association network constructed above. Then, the Gaussian interaction profile kernel similarity between microbe *m(i)* and *m(j)* is calculated from their interaction profiles as follows:

$$SM(m(i),m(j))=\exp(\text{-}\gamma_{m}\|m(i)\text{-}m(j){\|}^{2})$$ (1)

The parameter *γ*_*m*_ denotes the normalized kernel bandwidth, which is calculated based on the new kernel bandwidth parameter *γ*^*’*^_*m*_ as follows:

$$\gamma_{m}=\gamma_{m}^{'}/(\frac{1}{n_{m}}\sum_{k=1}^{n_{m}}{\left\|m(i)\right\|}^{2})$$ (2)

Here, *n*_*m*_ is the number of microbes and *γ*^*’*^_*m*_ is simply set to 1 [20].

### Disease similarity

Similar to microbes, Gaussian interaction profile kernel similarity between disease *d(i)* and *d(j)* can be defined as follows:

$$KSD(d(i),d(j))=\exp(\text{-}\gamma_{d}\|d(i)\text{-}d(j){\|}^{2})$$ (3)

$$\gamma_{d}=\gamma_{d}^{'}/(\frac{1}{n_{d}}\sum_{k=1}^{n_{d}}{\left\|d(i)\right\|}^{2})$$ (4)

where *n*_*d*_ is the number of diseases and *γ*^*’*^_*d*_ is also set to 1.

According to a prior study [37], similarity value ranges in [0, 0.3] are not informative, while similarity value ranges in [0.6, 1] are informative. To improve predictive accuracy, we regulate disease similarity by applying logistic function transformation. The function is defined as follows:

$$SD(d(i),d(j))=\frac{1}{1+e^{c\cdot KSD(d(i),d(j))+d}}$$ (5)

where *KSD(d(i)*,*d(j))* is the Gaussian interaction profile kernel similarity between diseases, and *c* and *d* are parameters that control the adjustment effect. For *KSD(d(i)*,*d(j))*∈[0,0.3], *SD(d(i)*,*d(j))*≈0; and for *KSD(d(i)*,*d(j))*∈[0.6,1], *SD(d(i)*,*d(j))*≈1. When *KSD(d(i)*,*d(j)) =* 0, we set *SD(d(i)*,*d(j)) =* 0.0001, which set *d* as log(9999). Vanunu et al. tune the parameter using cross validation and set *c =* -15 [37]. In the present study, we used the adjusted result, *SD*, to represent the final disease similarity.

### Construction of the heterogeneous network

**Fig 1:**
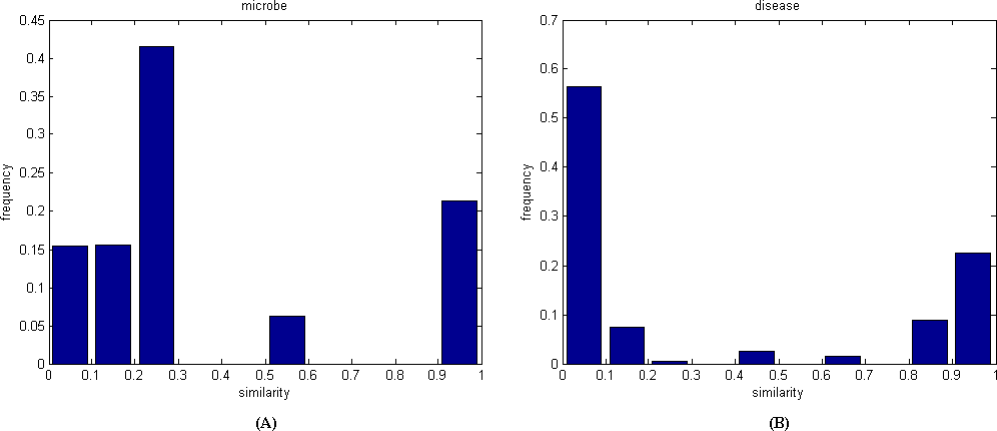
Frequency distribution of microbe similarity and disease similarity. (A) Frequency distribution of microbe similarity. (B) Frequency distribution of disease similarity.

Based on the microbe similarity and disease similarity calculated above, both the microbe similarity network and the disease similarity network can be constructed. In the microbe similarity network, let *M* = {*m(1)*, *m(2)*, …, *m(nm)*} denote the node set of *nm* microbes; the edge between two microbes is weighted by the similarity value of these two microbes. Likewise, in the disease similarity network, let *D* = {*d(1)*, *d(2)*, …, *d(nd)*} denote the node set of *nd* diseases, while the edge between two diseases is weighted by the similarity value of these two diseases. We further analyze the frequency distribution of edge weights in each network. Fig 1 indicates that the distribution of edge weights in the disease similarity network is more concentrated after logistic function transformation.

**Fig 2:**
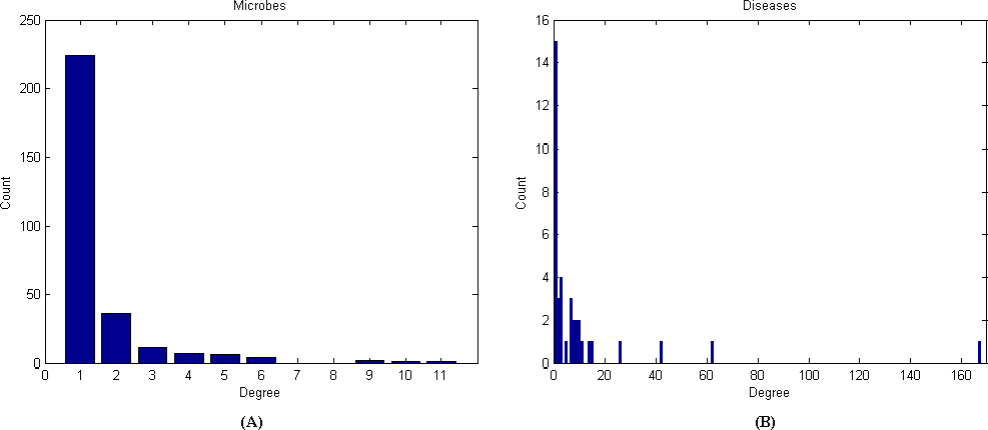
Degree distribution for microbes and diseases in the microbe-disease association network. (A) Degree distribution of microbes. (B) Degree distribution of diseases.

Besides, the microbe-disease association network can be modeled as a bipartite graph [38]. In the bipartite graph, the heterogeneous nodes correspond to either microbes or diseases, and edges denote the presence or absence of the associations between them. If there is a known association between disease *d(i)* and microbe *m(j)*, the weight of the edge is equal to 1; otherwise 0. To get a comprehensive view of the bipartite graph, we analyze the degree distribution of the microbes and diseases in the microbe-disease association network (Fig 2). It shows the activeness of all nodes in the entire network. On average, each microbe is associated with 1.54 diseases and each disease is associated with 11.54 microbes.

The global heterogeneous network contains above-mentioned two types of nodes (microbes and diseases) and three types of edges between them, which can be constructed by connecting the microbe similarity network and the disease similarity network via the known microbe-disease associations.

### BiRWHMDA

**Fig 3:**
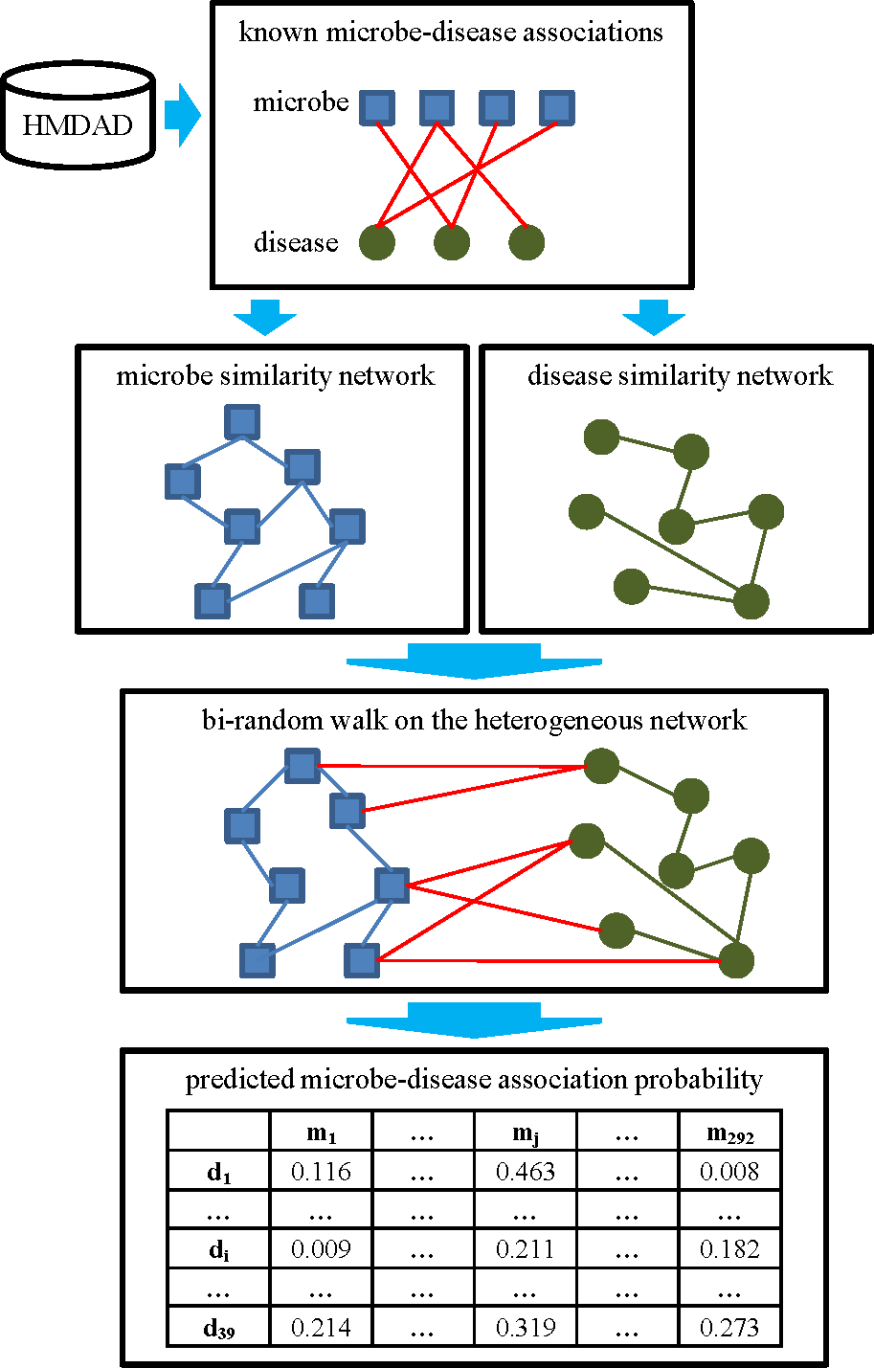
The flowchart of BiRWHMDA.

In this study, we developed a novel computational method of BiRWHMDA to predict human microbe-disease associations. Fig 3 shows the flowchart of BiRWHMDA. Firstly, microbe similarity and disease similarity could be calculated based on the known microbe-disease associations originated from HMDAD. Secondly, the global heterogeneous network was built by combining the microbe similarity network, the disease similarity network and the microbe-disease association network. Finally, the bi-random walk algorithm was performed on the heterogeneous network to obtain the association probability scores between microbes and diseases. The source code for BiRWHMDA is available in S2 File. In the following, we focus on the bi-random walk algorithm for microbe-disease association prediction.

**Fig 4:**
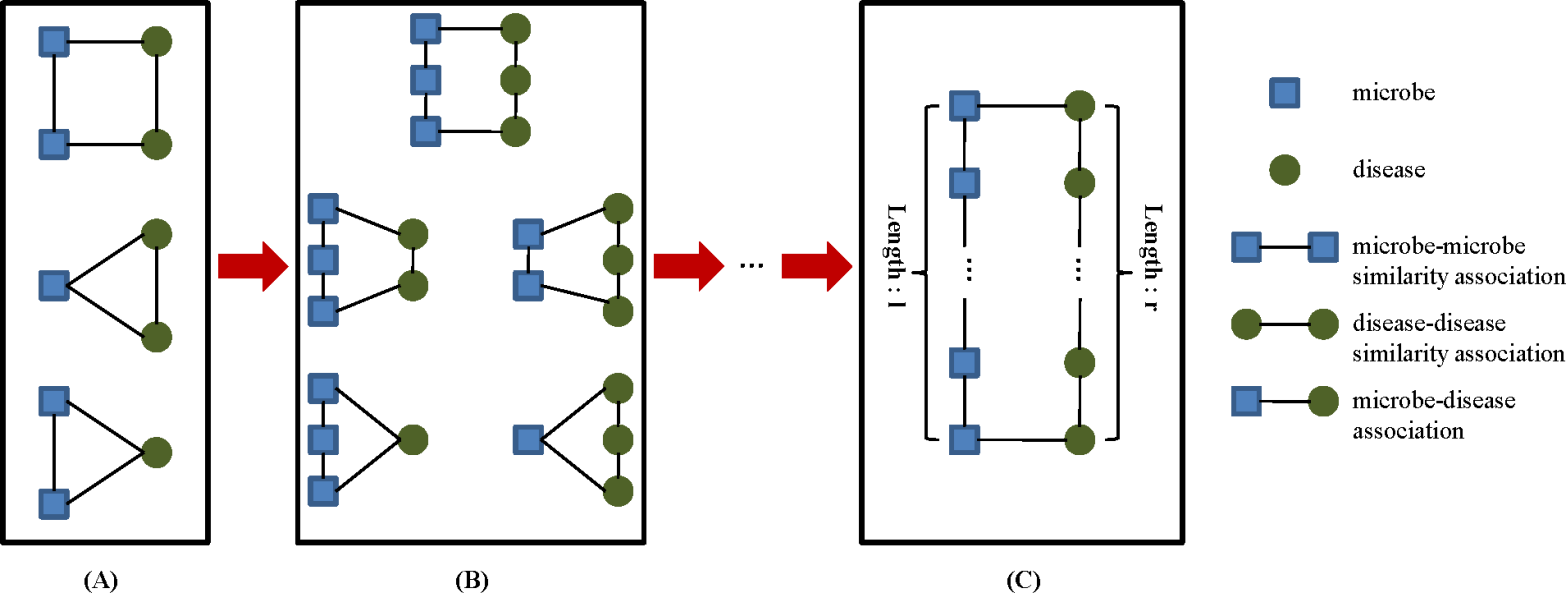
CBGs in the microbe-disease association network. (A) CBG of length 1. (B) CBG of length 2. (C) CBG of length max (*l*, *r*).

To have a deep understanding of this algorithm, we first introduce the concept of circular bigraph (CBG), which plays an important role in the procedure. A CBG is defined as a subgraph consisting of a microbe path {*m*_*1*_, *m*_*2*_, …, *m*_*m*_}and a disease path {*d*_*1*_, *d*_*2*_, …, *d*_*n*_}, with two ends linked by two known microbe-disease associations (*m*_*1*_, *d*_*1*_) and (*m*_*m*_, *d*_*n*_). The length of a CBG is defined as the length of the longer path of the two paths (Fig 4). A CBG describes a vicinity relation between the associations (*m*_*1*_, *d*_*1*_) and (*m*_*m*_, *d*_*n*_). Accordingly, a potential microbe-disease association is evaluated by its distance to other associations in the microbe similarity network and the disease similarity network [24].

Bi-random walk explores the CBG patterns by iteratively performing random walk on the microbe similarity network and the disease similarity network simultaneously, to infer novel microbe-disease associations [22]. The CBGs are weighted by a decay factor *α*, which ranges from 0 to 1; the importance of a CBG is decreased when the path length becomes longer. Nevertheless, the microbe similarity network and the disease similarity network contain diverse topologies and structures, which would generate disparate optimal amounts of random walk steps. To solve this problem, two parameters, *l* and *r*, are introduced to restrict steps on the two sides [26]. The iterative process is described as follows:

$$\mathrm{random}\;\mathrm{walk}\;\mathrm{on}\;\mathrm{the}\;\mathrm{microbe}\;\mathrm{similarity}\;\mathrm{network}:\;R_{m}=\alpha MD_{t-1}\cdot SM+\left(1-\alpha \right)A$$ (6)

$$\mathrm{random}\;\mathrm{walk}\;\mathrm{on}\;\mathrm{the}\;\mathrm{disease}\;\mathrm{similarity}\;\mathrm{network}:\;R_{d}=\alpha SD\cdot MD_{t-1}+\left(1-\alpha \right)A$$ (7)

**Fig 5:**
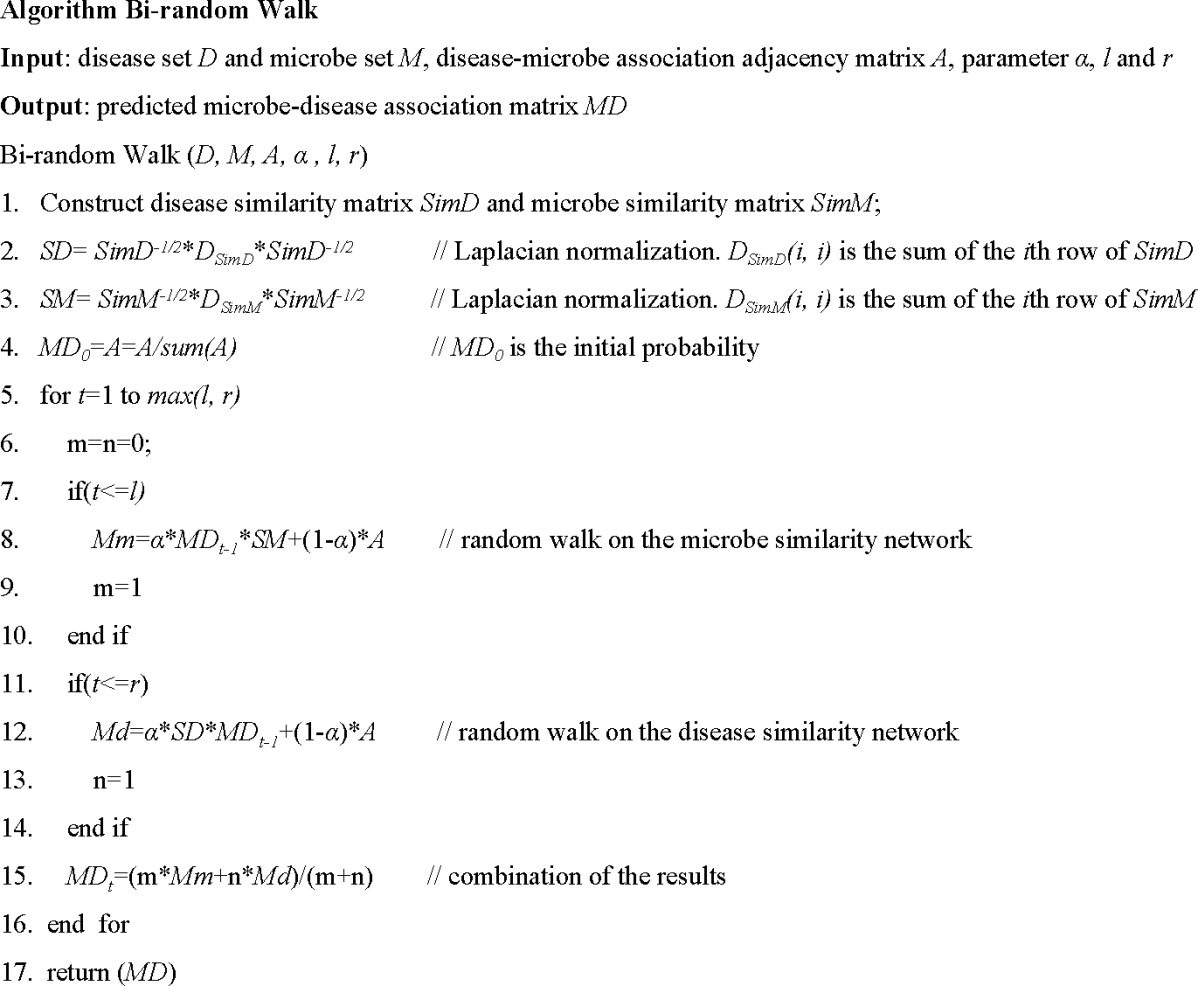
Description of algorithm bi-random walk.

Here, *α* is the decay factor. *R*_*d*_*(i*,*j)* and *R*_*m*_*(i*,*j)* denote the probability that disease *d(i)* associates with microbe *m(j)*. The algorithm is detailed in Fig 5:

At the end of the process, the matrix, *MD*, is acquired as the final prediction result, illustrating the association probability between each microbe and disease pair. For each disease, the potential associated microbes can be ranked according to the prediction probability scores. The top ranked microbes indicate the most relevant associations, potentially providing valuable information for further microbe-disease association research.

## Experiments and results

### Performance evaluation

To evaluate the prediction performance of the model we proposed, LOOCV and 5-fold cross validation were implemented on the 450 known microbe-disease associations. In each round of LOOCV, every known microbe-disease association was taken as the test sample, and the other known associations were taken as the training samples [39]. In addition, the microbe similarity and the disease similarity were recalculated at every turn. The predictive performance was evaluated by the rank of the test sample in the candidate samples (all unverified microbe-disease associations) based on their prediction scores. In 5-fold cross validation, the 450 known microbe-disease associations were randomly divided into five subsets. For each trial, one subset is processed as test samples and the other four subsets are processed as training samples; the unverified microbe-disease associations are regarded as candidate samples [40, 41]. Moreover, to reduce potential sample division bias, we performed random divisions 100 times.

**Fig 6:**
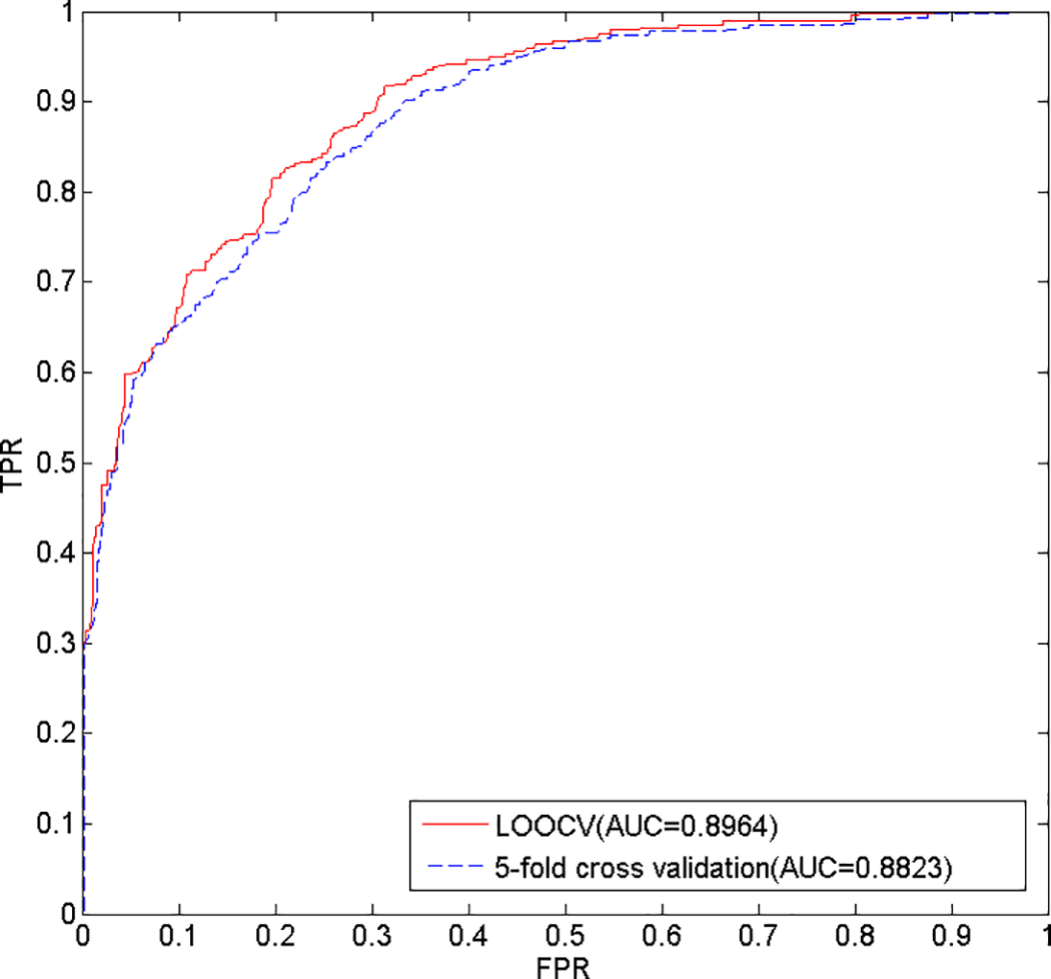
The ROC curve and AUC values of our method.

A receiver-operating characteristic (ROC) curve, which plots the relationship between the true positive rate (TPR, sensitivity) and the false positive rate (FPR, 1-specificity) by setting different thresholds, was applied to determine the prediction performance. Sensitivity represents the percentage of the test samples that rank higher than the given threshold, while specificity represents the opposite. AUC was also calculated, such that an AUC value of 1 denotes perfect performance, and an AUC value of 0.5 indicates random performance [42–44]. As a result, our model achieves AUC values of 0.8964 and 0.8808 in the LOOCV and 5-fold cross validation frameworks, respectively (Fig 6). While 0.8808 is the average AUC value of 100 operations in 5-fold cross validation, we further obtain the standard deviation of 0.0029. Ultimately, these results confirm the superior performance of this method.

### Effect of parameters

There are three parameters in our model. The parameter *α* is the decay factor, which is used to down-weights the importance of a CBG when its path becomes longer. The parameters *l* and *r* are introduced to limit the number of random walk steps in the microbe and disease similarity network, respectively. To investigate the effects of the three parameters, we set various values for them and then calculated the AUC values by LOOCV. The details can be seen in Table 1. The experimental results illustrate that BiRWHMDA achieves satisfactory performance when parameter *l* is equal to *r*. Taking various parameter combinations into account, we set the three parameters as *α =* 0.4, *l =* 2 and *r =* 2 in our experiment.

**Table 1:** Effect of parameters *α*, *l* and *r* in the results.

***α* = 0.2**				
	***r* = 1**	***r* = 2**	***r* = 3**	***r* = 4**
***l* = 1**	0.8944	0.8631	0.7892	0.7275
***l* = 2**	0.8612	0.8952	0.8656	0.7911
***l* = 3**	0.8530	0.8610	0.8954	0.8658
***l* = 4**	0.8527	0.8529	0.8610	0.8954
***α* = 0.4**				
	***r* = 1**	***r* = 2**	***r* = 3**	***r* = 4**
***l* = 1**	0.8944	0.8807	0.8424	0.7930
***l* = 2**	0.8669	0.8964	0.8820	0.8480
***l* = 3**	0.8513	0.8653	0.8960	0.8819
***l* = 4**	0.8503	0.8511	0.8647	0.8916
***α* = 0.6**				
	***r* = 1**	***r* = 2**	***r* = 3**	***r* = 4**
***l* = 1**	0.8944	0.8880	0.8676	0.8416
***l* = 2**	0.8700	0.8966	0.8895	0.8660
***l* = 3**	0.8492	0.8670	0.8965	0.8885
***l* = 4**	0.8478	0.8483	0.8648	0.8960
***α* = 0.8**				
	***r* = 1**	***r* = 2**	***r* = 3**	***r* = 4**
***l* = 1**	0.8944	0.8930	0.8805	0.8623
***l* = 2**	0.8727	0.8969	0.8917	0.8747
***l* = 3**	0.8467	0.8667	0.8956	0.8817
***l* = 4**	0.8425	0.8428	0.8580	0.8636

### Comparison with other methods

To our knowledge, RWRHMDA, KATZHMD and PBHMDA are state-of-the-art computational methods for predicting microbe-disease associations. In considering important differences, RWRHMDA is based on a stochastic process that aims to predict candidate microbes for a disease by calculating the probability of the random walker reaching them [19]; KATZHMDA is based on the KATZ measure that calculates nodes’ similarity in the heterogeneous network to solve the problem of link prediction [20]; PBHMDA is a path-based method that utilizes a special depth-first search algorithm in the heterogeneous interlinked network to infer potential microbe-disease associations [21]. These methods are similar in that they are all accomplished based on a heterogeneous network which is constructed by connecting the microbe similarity network and the disease similarity network via the known microbe-disease associations.

Our method, BiRWHMDA, aims to predict novel microbe-disease associations by capturing CBG patterns on the global heterogeneous network. It is a multi-task learning method, which explores the missing microbe-disease associations simultaneously, instead of prioritizing candidate microbes for a specific disease [45–47]. Additionally, BiRWHMDA can predict novel microbes for diseases without any known associated microbe information.

**Fig 7:**
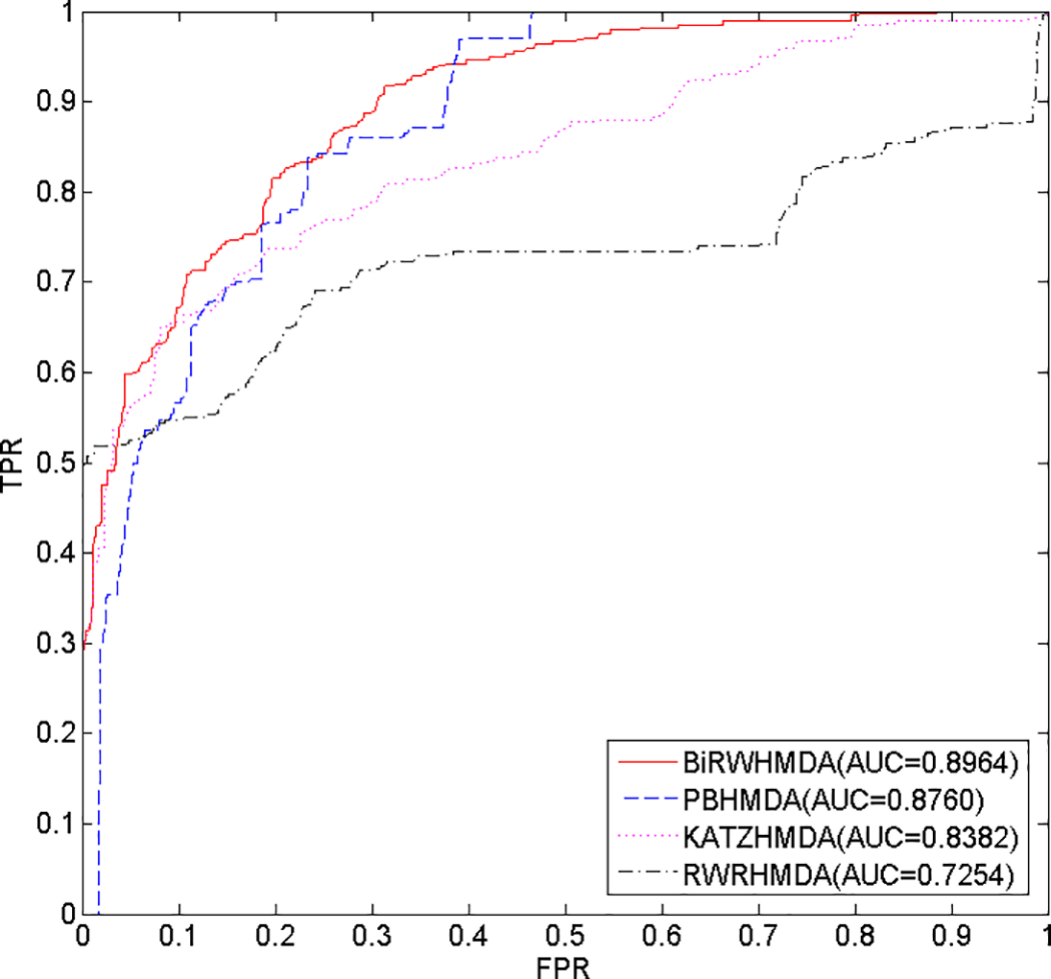
The ROC curve and AUC values of different methods.

In this study, we implemented these three methods using the same datasets as BiRWHMDA, and then compared their performance by the LOOCV method. Consequently, BiRWHMDA achieves the best performance among all the methods with an AUC value of 0.8964, while RWRHMDA, KATZHMDA and PBHMDA yield AUC values of 0.7254, 0.8382 and 0.8760, respectively (Fig 7). The results demonstrate that BiRWHMDA works better than the other methods, and the predictive performance of BiRWHMDA increases nearly two percentage points higher than the latest method, PBHMDA.

### Case studies

We also implemented case studies involving asthma and inflammatory bowel disease (IBD) to further evaluate the ability of our method to predict novel microbe-disease associations. Here, novel associations refer to the microbe-disease pairs that are not known to be associated in the dataset. For each disease, the candidate associated microbes are ranked according to the prediction association scores obtained from BiRWHMDA. We observed microbes from the top 10 candidate microbes confirmed by current research. Furthermore, we compare the results of BiRWHMDA with the latest method, PBHMDA. In this study, we assume that if a microbe is associated with one disease, the genus that the microorganism belongs to is also associated with the disease.

Asthma is a common long-term inflammatory disease of the lung airways. In BiRWHMDA, a total of eight of the predicted microbes in the top 10 candidate microbes have been validated (Table 2). Pseudomonas aeruginosa could cause asthma, which has already been diagnosed by bronchoscopic examination [48]. Lactobacillus rhamnosus is associated with asthma prevention [49]. Colonization by clostridium difficile at 1 month of age is associated with the incidence of asthma between ages 6 and 7 [50]. Firmicutes and actinobacteria are present in lower proportions in asthmatic patients [51]. Clostridium coccoides XIVa species is significantly associated with a positive Asthma Predictive Index (API) [52]. Propionibacterium acnes is more prevalent in asthma patients; therefore, Propionibacterium is also considered to be associated with asthma [53]. Only Burkholderia and Oxalobacter formigenes have not been validated to date. The top 10 candidate microbes of asthma obtained from PBHMDA are also listed in Table 2; nine of these microbes have been previously confirmed [49, 51, 54–59].

**Table 2:** Prediction results of associated microbes for disease asthma.

Rank	BiRWHMDA		PBHMDA	
	Microbe	Evidence	Microbe	Evidence
1	Pseudomonas	PMID:13268970	Firmicutes	PMID:23265859
2	Lactobacillus	PMID:20592920	Lactobacillus	PMID:20592920
3	Burkholderia	Unconfirmed	Lachnospiraceae	Lee et al., 2014
4	Clostridium difficile	PMID:21872915	Veillonella	PMID:25329665
5	Firmicutes	PMID:23265859	Bacteroides	PMID:18822123
6	Actinobacteria	PMID:23265859	Bacteroidaceae	Qiu et al., 2013
7	Clostridium coccoides	PMID:21477358	Streptococcus	PMID:17950502
8	Propionibacterium	PMID:27433177	Fusobacterium	Dang et al., 2013
9	Propionibacterium acnes	PMID:27433177	Actinobacteria	PMID:23265859
10	Oxalobacter formigenes	Unconfirmed	Eubacterium	unconfirmed

IBD is a group of inflammatory conditions of the colon and small intestine. In BiRWHMDA, each of the microbes in the top 10 has been validated (Table 3). There is an inverse association between helicobacter pylori and IBD [60]. Research shows a significant relationship between the simultaneous presence of toxigenic strains of staphylococcus aureus and clostridium difficile in IBD patients; staphylococcus is thus validated [14]. Clostridium coccoides are less represented in A-IBD patients [61]. Bacteroidetes, firmicutes, Prevotella and clostridia have been shown to be associated with IBD via the Kruskal-Wallis test [62]. Bifidobacterium shows an increased proportion in IBD [63]. The top 10 candidate microbes for IBD obtained from PBHMDA are also listed in Table 3; of these, eight microbes have been previously validated [61, 62, 64–66].

**Table 3:** Prediction results of associated microbes for disease IBD.

Rank	BiRWHMDA		PBHMDA	
	Microbe	Evidence	Microbe	Evidence
1	Helicobacter pylori	PMID:22221289	Bacteroidetes	PMID:25307765
2	Clostridium difficile	Azimirad et al.,2012	Firmicutes	PMID:25307765
3	Clostridium coccoides	PMID:19235886	Veillonella	unconfirmed
4	Bacteroidetes	PMID:25307765	Prevotella	PMID:25307765
5	Firmicutes	PMID:25307765	Haemophilus	unconfirmed
6	Prevotella	PMID:25307765	Bacteroidaceae	Maukonen et al.,2009
7	Staphylococcus aureus	Azimirad et al.,2012	Lactobacillus	PMID:26340825 26340825
8	Bifidobacterium	PMID:24478468	Bacteroides	Maukonen et al.,2009
9	Staphylococcus	Azimirad et al.,2012	Clostridium coccoides	PMID:19235886
10	Clostridia	PMID:25307765	Streptococcus	PMID:23679203

In summary, these case studies further demonstrate that the approach we proposed is powerful in predicting novel microbe-disease associations. The predictions for all the 39 diseases are listed in S3 File.

## Conclusion

A growing body of research suggests that the microbiome plays a vital role in human health and disease. Microbe-disease associations can not only reveal disease pathogenesis but also contribute to disease diagnosis and prognosis [67]. Nevertheless, due to the limited research on existing microbe-disease association data, only a few methods have been developed to address the gap.

In the present study, we proposed a novel approach based on bi-random walk on the heterogeneous network to predict novel microbe-disease associations. The heterogeneous network is constructed by connecting the microbe similarity network and the disease similarity network via the known disease-microbe associations. The measure we utilized to calculate microbe similarity and disease similarity was the Gaussian interaction profile kernel similarity measure. In addition, a logistic function was applied to adjust disease similarity. We sought to obtain the predictive association scores between each microbe and disease pair through BiRWHMDA. For each disease, the top ranked microbes are considered the most probable associated microbes. Cross validation frameworks, including LOOCV and 5-fold cross validation, were also implemented to evaluate predictive performance of our approach. Moreover, the approach was compared with three other state-of-the-art methods by using LOOCV. Ultimately, our method obtained better performance than these competing methods. Additionally, we implemented case studies for asthma and IBD to evaluate the predictive performance of BiRWHMDA. In total, eight and ten of the predicted microbes in the top 10 microbe candidates have been confirmed by recent studies. Our method demonstrated favorable utility in predicting novel microbe-disease associations.

Despite the current success, there are still some limitations that can be improved in future studies. First, only one database exists: the HMDAD, which contains 483 verified microbe-disease association records. Therefore, predictive performance will be certainly limited due to the lack of available experimental data. This could be solved through an increase in microbe-disease associations discovered in the future. In addition, microbe and disease similarity are calculated based solely on known microbe-disease associations, which could cause bias for microbes and diseases already extant in the database. Data from different sources should be integrated to improve the completeness and quality of the experimental data, which would ultimately be conductive to improving predictive performance.

## Supporting information

S1 File The dataset explored in this work. (ZIP)

S2 File The source code for BiRWHMDA. (ZIP)

S3 File The prediction results for each disease. (ZIP)
